# Un cas de phéochromocytome découvert au cours d’une grossesse gémellaire: un diagnostic à ne pas méconnaître et revue de littérature

**DOI:** 10.11604/pamj.2018.29.168.13901

**Published:** 2018-03-23

**Authors:** Bahia Habra, Ghizlane El Mghari, Nawal El Ansari

**Affiliations:** 1 Service d’Endocrinologie, Diabétologie et Maladies Métaboliques, Hôpital Arrazi, CHU Mohammed VI, Laboratoire de Recherche de Pneumo-Cardio-Immunopathologie et Métabolisme (PCIM), Faculté de Médecine et de Pharmacie de Marrakech, Université Cadi Ayad, Marrakech, Maroc

**Keywords:** Phéochromocytome, grossesse gémellaire, pathologie rare, Gemellar pregnancy, phéochromocytoma, pathology

## Abstract

Le phéochromocytome est une pathologie rare, potentiellement grave. Dans la littérature, moins de 250 cas sont décrits. La fréquence des diagnostics méconnus pendant la grossesse est expliquée par la rareté de cette association et la similitude avec l'hypertension gravidique. Le pronostic lié à la précocité du diagnostic et une prise en charge pluridisciplinaire. D'où l'intérêt d'explorer toutes les hypertensions artérielles mal définies ou à caractère familial au cours de la grossesse. La certitude diagnostic est menée par des tests biologiques, un bilan de localisation par échographie ou par imagerie par résonance magnétique (IRM). Une préparation médicale permet de choisir selon le terme de retirer la tumeur avant ou après l'accouchement. Nous rapportant un cas de phéochromocytome diagnostiqué au cours d'une grossesse gémellaire de 26 semaines d'aménorrhée (SA). Nous rapportant les éléments du diagnostic clinique et biologique de l'imagerie, du traitement et du pronostic vital maternel et fœtal.

## Abstract (en)

The pheochromocytoma is a rare, but potentially serious, which can be revealed by pregnancy. Less than 250 cases described in the literature .The rarity of this association and similarity with pregnancy-induced hypertension explains the frequency of unknown diagnoses during pregnancy. A privileged time of discovery, the prognosis associated with early diagnosis, a multidisciplinary management. Hence the need to explore all arterial hypertension poorly defined or family nature during pregnancy. The certainty diagnosis is conducted by biological tests provided are thought of, a review of localization by ultrasound or magnetic resonance imaging (MRI), a medical preparation can choose according to the term to remove the tumor before or after delivery. We related a case of pheochromocytoma diagnosed during a twin pregnancy of 26 weeks of amenorrhea (SA), bringing us the elements of the clinical and laboratory diagnostic imaging, treatment and maternal and fetal prognosis.

## Introduction

Le phéochromocytome est une pathologie rare, potentiellement grave, qui peut être révélée par la grossesse. La rareté de cette association et la similitude avec l'hypertension gravidique explique la fréquence des diagnostics méconnus pendant la grossesse. Le pronostic maternel et fœtal est conditionné par un diagnostic précoce et une prise en charge médicale multidisciplinaire qui prépare à l´exérèse de la tumeur. La certitude diagnostique est donnée par des tests biologiques qui sont simples et fiables à condition d´y penser, la tumeur est localisée par échographie ou par imagerie par résonance magnétique (IRM). La préparation par *-bloquant permet de choisir, en fonction du terme, de retirer la tumeur avant ou après l´accouchement. Cette stratégie permet de réduire une mortalité maternelle et fœtale qui est très élevée quand le diagnostic est méconnu.

## Patient et observation

**Figure 1:**
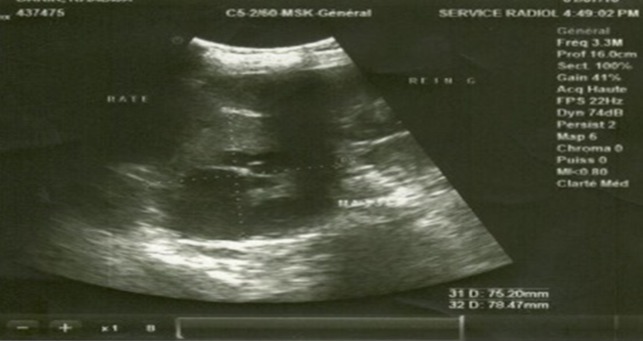
Echographie abdominale: masse inter-splénorénale hétérogène mesurant 75x78 mm, de forme arrondie, siège de zone kystique

**Figure 2:**
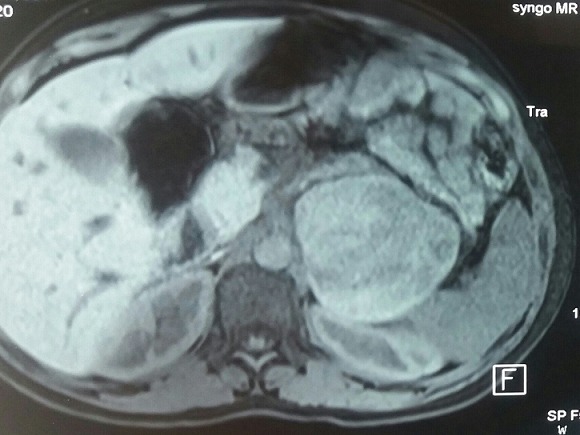
l’IRM abdominale sans injection de produit de contraste au cours de la grossesse: Processus tumoral sur la loge surrénalienne gauche solidokystique cde 7,5x8,5 cm faisant évoquer un phéochromocytome associés à des nodules hépatiques probablement secondaire

**Figure 3:**
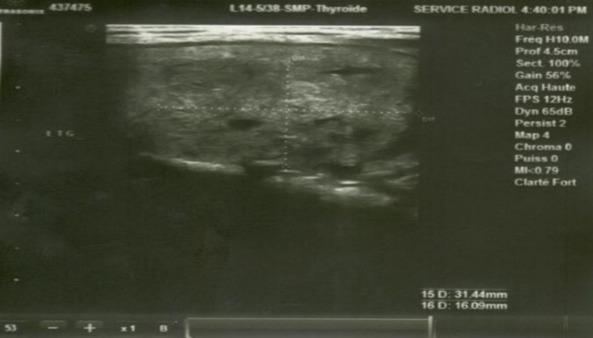
Echographie cervicale: Goitre multihétéronodulaire TI-RADS 4B, avec un Nodule lobaire gauche: 13x16 mm hypo écho gène hétérogène, contours flous micro calcification

patiente K.D âgée de 32 ans , ayant un frère hypertendu, mère de 4 enfants, les accouchements antérieurs par voix basse sans incidents, qui a présenté depuis 1 an et demi des palpitation avec sueurs nocturnes, pas de chiffres tensionnels élevés sans explorations .Par la suite, la patiente s'est présentée en consultation , enceinte d'une grossesse gémellaire de 18 SA , le diagnostic de phéochromocytrome fut évoqué devant une triade de Ménard avec des Pics tensionnels à 200/100 mmhg, sans œdèmes des membres inferieures sans protéinurie avec crampes musculaires, paresthésies, bouffées de chaleurs, est confirmé par le bilan biologique et radiologique (Tableau 1, (Figure 1,Figure 2, Figure 3), l'examen obstétrical était normal sans hypotrophie fœtal , un traitement par methyldopa fut remplacé par la suite, par prazozine permit de normaliser la tension artériel(TA). Une concertation multidisciplinaire va aboutir à la programmation à 34 SA d'une césarienne sous anesthésie générale, la surrénalectomie gauche soit en un seul temps soit en 2 temps, la corticothérapie de maturation pulmonaire a été indiquée (bétaméthasone, Célestène^®^ 12 mg). Cependant, en raison d'une rupture prématurée de la poche des eaux à 33 SA, l'intervention par césarienne fut décidée à ce terme sous anesthésie générale ,permit la naissance d'un premier jumeaux vivant de sexe féminin de 2160 g qui a séjournée en reanimation pédiatrique pendant 7 jours, avec un décès néonatal du deuxième jumeaux de sexe masculin, des pics hypertensives qui se sont normalisés à J7 du postpartum après le remise sous alpha bloquant, à la 6 semaines de l'accouchement ,une TDM surrénalienne réalisée avec injection du produit de contraste montrant discrète augmentation du processus surrénalien gauche mesurant 10x9x8 cm arrondie de densité spontanée siège de zone kystique multi lobulé, refoule en haut le queue du pancréas au contact intime avec la veine splénique , après préparation de la malade , l'exérèse du phéochromocytome réalisée par voie sous-costale sans complications . Les suites post-opératoires immédiates ont été simples. Un dosage des dérivés méthoxylés des catécholamines urinaires a permis d'exclure a priori l'existence d'une localisation ectopique ou secondaire du phéochromocytome. Les symptômes cliniques ont totalement régressé. L'analyse anatomopathologique de la pièce opératoire a confirmé la nature de la tumeur avec un score 4 de PASS, absence d'effraction capsulaire, une exploration scintigraphie à la MIBG, une cytoponction du nodule thyroïdien TI-RADS 4B et notamment un suivi régulier sont prévus

**Tableau 1:** Tableau de bilan biologique et radiologique

Bilan biologique et radiologique	Résultats
**Dosage des Métanéphrines urinaires**	
Normétanéphrine	19 fois la normale
Métanéphrine	96 fois la normale
**Bilan de NEM**	
PTH	Normale
Bilan phosphocalcique	Normal
Calcitonine	Normale
Vitamine D	carence
Bilan thyroidfien	Normal
La recherche d’anticorps (anticorps anti-thyropéroxydase, anticorps antirécepteurs de la TSH)	En cours
Echographie abdominale (figure 1)	Masse inter-splénorénale hétérogène mesurant 75x78 mm, de forme arrondie, siège de zone kystique
L’IRM abdominale sans injection de produit de contraste au cours de la grossesse (figure 2)	Processus tumoral sur la loge surrénalienne gauche solidokystique cde 7,5x8,5 cm faisant évoquer un phéochromocytome associés à des nodules hépatiques probablement secondaire
Echographie cervicale (figure3)	Goitre multihétéronodulaire TI-RADS 4B, avec un Nodule lobaire gauche: 13x16 mm hypo écho gène hétérogène, contours flous micro calcification

## Discussion

Dans la littérature, le phéochromocytome est une tumeur rare dont la fréquence atteint à peine 1/10000 cas dans la population générale, et touche 0,5 à 1 % des hypertendus. L'incidence est actuellement voisine de 1/50000 à 1/54000 chez la femme enceinte (chiffres françaises) [1,2]. Deux cent cinquante cas survenus au cours d'une grossesse ont été diagnostiqués et publiés avant 2002, dont 85 % pris en charge avant l'accouchement. En comparaison, l'incidence de l'HTA gravidique est de 10 %, celle de la prééclampsie de 1 % alors que l'HTA chronique concerne 1 % à 5 % des grossesses (parmi lesquelles on compte le phéochromocytome) La date de mise en évidence de la pathologie détermine le pronostic materno-fœtal. Il existe une surmortalité importante en cas de diagnostic tardif, puisque les chiffres sont respectivement de 19 % et 25 % pour la mortalité maternelle et fœtale en l'absence de diagnostic, contre 1 % et 12 % en cas de traitement précoce [3,4]. Le diagnostic repose sur la clinique(signes d'appels) la biologie(diagnostic positif), et la radiologie(diagnostic topographique), la difficulté du diagnostic tient de sa rareté , Le phéochromocytome diffère de l´HTA gravidique par la présence de la triade symptomatique céphalées-sueurs-palpitations qui a une sensibilité de 90 % [5,6] et par l´absence de signes rénaux (protéinurie, hyperuricémie), C´est le dosage des métanéphrines urinaires totales qui offre la meilleure sensibilité pour le diagnostic de phéochromocytome [5] La grossesse ne modifie pas les valeurs de ces paramètres. Le premier traitement à instaurer est un α-bloquant (prazozine, phénoxybenzamine), dont le rôle est de contrôler la tension artérielle et de prévenir les complications qui lui sont liées en limitant l'effet de la noradrénaline (α agoniste). La prazozine est prescrite à dose progressive de 0,25 mg à 5 mg 2 fois/j, au risque de provoquer un collapsus par effet première dose. Le second, un β-bloquant, dont l'instauration doit obligatoirement se faire après et en coprescription du précédent, prévient ou traite une tachycardie ou un trouble du rythme démasqué par les α-bloquants. L'adjonction du β-bloquant n'est pas systématique et ne s'applique qu'aux patientes qui tolèrent mal leur tachycardie, présentent des troubles du rythme ou à celles dont le phéochromocytome sécrète principalement de l'adrénaline. L´anesthésie doit être confiée à une équipe entraînée pour obtenir un contrôle de l´excès de catécholamines circulantes. La date opératoire diffère suivant que l´on se trouve avant ou après 24 SA [7]. Avant 24 SA la tumeur doit être réséquée au plus vite, malgré le risque de fausse couche même tardive, car des crises catécholergiques aux conséquences plus graves peuvent survenir sous alpha-bloquants. Après 24 SA, l´intervention est retardée du fait de l´utérus gravide. On attend (au mieux) la maturité pulmonaire pour faire une césarienne par voie médiane, suivie d´une exérèse tumorale au cours de la même anesthésie générale [8].

## Conclusion

Il s'agit d'une pathologie rare pour laquelle la grossesse constitue une période privilégiée de découverte, du fait de l'étendue de la population prise en charge. Toute HTA au cours de la grossesse n´est pas forcément une HTA gravidique L´association à la triade céphalées-sueurs-palpitations doit faire évoquer le phéochromocytome .Son pronostic est directement lié à la précocité du diagnostic ainsi qu'à une prise en charge pluridisciplinaire adaptée. Lorsque le phéochromocytome est découvert au deuxième ou au troisième trimestre d'une grossesse, l'ensemble des auteurs recommande de programmer un accouchement par césarienne après préparation médicale, et d'y associer selon les équipes l'ablation de la tumeur. L'évocation de cette pathologie est plus difficile que son diagnostic, du fait de la fréquence des tableaux incomplets et aspécifiques qui doivent inciter les praticiens à une exploration systématique de toutes les hypertensions artérielles mal définies ou à caractère familial au cours de la grossesse.

## Conflits d’intérêts

Les auteurs ne déclarent aucun conflit d'intérêts.
